# Response to: Arora, V.; Gupta, N.K.; Nath, D.K.; Tandan, A.; Chandra, P. Comments on “The Dental Aesthetic Index and Dental Health Component of the Index of Orthodontic Treatment Need as Tools in Epidemiological Studies”. *Int. J. Environ. Res. Public Health* 2011, *8*, 3277–3286

**DOI:** 10.3390/ijerph9093283

**Published:** 2012-09-07

**Authors:** Chrystiane F. Cardoso, Alexandre F. Drummond, Elisabeth M.B. Lages, Henrique Pretti, Efigênia F. Ferreira, Mauro Henrique N.G. Abreu

**Affiliations:** 1 Department of Community and Preventive Dentistry, Universidade Federal de Minas Gerais, Avenida Antônio Carlos, 6627–ZIP code 31270.901 sala 3304, Belo Horizonte, MG, Brazil; Email: chrys@nwnet.com.br (C.F.C.); efigeniaf@gmail.com (E.F.F.); 2 Department of Paediatric Dentistry and Orthodontics, Universidade Federal de Minas Gerais, Avenida Antônio Carlos, 6627–ZIP Code 31270.901 sala 3304, Belo Horizonte, MG, Brazil; Email: afdorto@googlemail.com (A.F.D.); bethlages@uai.com.br (E.M.B.L.); hpretti@uai.com.br (H.P.)

In response to the Commentary submitted by Arora *et al.* [1], which has questioned some methodological issues in our article [2], we would like to respond item by item.

(1) Arora *et al.* have pointed out that we performed a study with a prevalence of orthodontic treatment need (according to the gold standard) of 91%. However, according to Table 4 of our study, the real prevalence of orthodontic treatment need is **52%**.Despite the fact that we chose the archives of Specialization Course in Orthodontics of a Dental School, this high prevalence (52%) is similar to the results of other studies **because these validation studies are usually conducted in orthodontics settings**, where study models are provided and where most cases for treatment are due to the need for diagnosis [3,4,5,6]. It is important to reinforce that we have also discussed these points in the second paragraph of page 3283.
(2) Arora *et al.* questioned “*whether 9% casts shown to be not requiring orthodontic treatment were actually of subjects who did not require orthodontic treatment?*” The models evaluated (n = 131) were a sample of 198 models of oral cavity of all orthodontic patients from Universidade Federal de Minas Gerais (first paragraph, page 3279). These models pertained to patients who required orthodontic treatment in this University. The clinical evaluation of orthodontic treatment need is a complex process which involves radiograph evaluation, facial evaluation, dental evaluation, and desires [7]. The gold standard of orthodontic treatment need in our study was determined by a consensus of three experts. These experts have evaluated dental models. This evaluation is quite different to a clinical orthodontic diagnosis. So, the prevalence of patients without orthodontic need (48%) was not a surprise for us.
(3) Considering that the prevalence of orthodontic treatment need was not 91%, as pointed out by Arora *et al.*, this simulation of accuracy is not relevant.
(4) To evaluate reproducibility we examined 13 models. In the literature concerning reproducibility of occlusal indexes there is no unique option regarding sample size. Beglin *et al.* [5] and Firestone *et al.* [8] evaluated 40 dental models in order to measure reproducibility. Ovsenik and Primoziv [3] on the other hand studied 10 models. In this regard, our methodology lies between these extremes presented in the literature.
(5) The last issue is related to the validity evaluation. The ideal value of an Area Under Curve (AUC) is 1.0. The statistical program that we have used measured the AUC for both indexes and the 95% confidence intervals (95% CI). These confidence intervals indicated if the curve crosses the non-significant 0.5 value at any point. Considering that 95% CI for both indexes did not cross the non-significant 0.5 value, we sustain that both indexes present reasonable (not perfect) accuracy [9].

It is important to reinforce that we discuss the limitations of our study in the last paragraph, page 3284. However, we cannot accept the criticisms laid out in this Commentary. 
